# Supplementary material for: Roscovitine, a CDK Inhibitor, Reduced Neuronal Toxicity of mHTT by Targeting HTT Phosphorylation at S1181 and S1201 In Vitro
Source: Int J Mol Sci. 2024 Nov 16;25(22):12315. doi: 10.3390/ijms252212315 (PMC11594617; doi:10.3390/ijms252212315)
Supplement: Supplementary file 1 [file ijms-25-12315-s001.zip › ijms-3296681-supplementary.pdf]

Roscovitine, a CDK Inhibitor, Reduced Neuronal Toxicity of mHTT by Targeting HTT Phosphorylation at S1181 and S1201 In Vitro

Supplement materials.

S1. Supplement information.

A list of kinases included in the library screening.

Kinase Name	Kinase Name	Kinase Name	Kinase Name
ABL1	ABL2/ARG	ACK1	AKT1
AKT2	AKT3	ALK	ALK1/ACVRL1
ALK2/ACVR1	ALK3/BMPR1A	ALK4/ACVR1B	ALK5/TGFBR1
ALK6/BMPR1B	ARAF	ARK5/NUAK1	ASK1/MAP3K5
Aurora A	Aurora B	Aurora C	AXL
BLK	BMPR2	BMX/ETK	BRAF
BRK	BRSK1	BRSK2	BTK
CAMK1a	CAMK1b	CAMK1d	CAMK1g
CAMK2a	CAMK2b	CAMK2d	CAMK2g
CAMK4	CAMKK1	CAMKK2	CDC7/DBF4
CDK1/cyclin A	CDK1/cyclin B	CDK1/cyclin E	CDK14/cyclin Y (PFTK1)
CDK16/cyclin Y (PCTAIRE)	CDK17/cyclin Y (PCTK2)	CDK18/cyclin Y (PCTK3)	CDK19/cyclin C
CDK2/cyclin A	CDK2/Cyclin A1	CDK2/cyclin E	CDK2/cyclin O
CDK3/cyclin E	CDK4/cyclin D1	CDK4/cyclin D3	CDK5/p25
CDK5/p35	CDK6/cyclin D1	CDK6/cyclin D3	CDK7/cyclin H
CDK9/cyclin K	CDK9/cyclin T1	CDK9/cyclin T2	CHK1
CHK2	CK1a1	CK1a1L	CK1d
CK1epsilon	CK1g1	CK1g2	CK1g3
CK2a	CK2a2	c-Kit	CLK1
CLK2	CLK3	CLK4	c-MER
c-MET	COT1/MAP3K8	CSK	c-Src
CTK/MATK	DAPK1	DAPK2	DCAMKL1
DCAMKL2	DDR1	DDR2	DLK/MAP3K12
DMPK	DMPK2	DRAK1/STK17A	DYRK1/DYRK1A
DYRK1B	DYRK2	DYRK3	DYRK4
EGFR	EPHA1	EPHA2	EPHA3
EPHA4	EPHA5	EPHA6	EPHA7
EPHA8	EPHB1	EPHB2	EPHB3
EPHB4	ERBB2/HER2	ERBB4/HER4	ERK1
ERK2/MAPK1	ERK5/MAPK7	ERK7/MAPK15	ERN1/IRE1

Kinase Name	Kinase Name	Kinase Name	Kinase Name
ERN2/IRE2	FAK/PTK2	FER	FES/FPS
FGFR1	FGFR2	FGFR3	FGFR4
FGR	FLT1/VEGFR1	FLT3	FLT4/VEGFR3
FMS	FRK/PTK5	FYN	GCK/MAP4K2
GLK/MAP4K3	GRK1	GRK2	GRK3
GRK4	GRK5	GRK6	GRK7
GSK3a	GSK3b	Haspin	HCK
HGK/MAP4K4	HIPK1	HIPK2	HIPK3
HIPK4	HPK1/MAP4K1	IGF1R	IKKa/CHUK
IKKb/IKBKB	IKKe/IKBKE	IR	IRAK1
IRAK4	IRR/INSRR	ITK	JAK1
JAK2	JAK3	JNK1	JNK2
JNK3	KDR/VEGFR2	KHS/MAP4K5	KSR1
KSR2	LATS1	LATS2	LCK
LCK2/ICK	LIMK1	LIMK2	LKB1
LOK/STK10	LRRK2	LYN	LYN B
MAK	MAPKAPK2	MAPKAPK3	MAPKAPK5/PRAK
MARK1	MARK2/PAR-1Ba	MARK3	MARK4
MEK1	MEK2	MEK3	MEK5
MEKK1	MEKK2	MEKK3	MEKK6
MELK	MINK/MINK1	MKK4	MKK6
MKK7	MLCK/MYLK	MLCK2/MYLK2	MLK1/MAP3K9
MLK2/MAP3K10	MLK3/MAP3K11	MLK4	MNK2
MRCKa/CDC42BPA	MRCKb/CDC42BPB	MSK1/RPS6KA5	MSK2/RPS6KA4
MSSK1/STK23	MST1/STK4	MST2/STK3	MST3/STK24
MST4	MUSK	MYLK3	MYLK4
MYO3A	MYO3b	NEK1	NEK11
NEK2	NEK3	NEK4	NEK5
NEK6	NEK7	NEK8	NEK9
NIM1	NLK	OSR1/OXSR1	P38a/MAPK14
P38b/MAPK11	P38d/MAPK13	P38g	p70S6K/RPS6KB1
p70S6Kb/RPS6KB2	PAK1	PAK2	PAK3
PAK4	PAK5	PAK6	PASK
PBK/TOPK	PDGFRa	PDGFRb	PDK1/PDPK1
PEAK1	PHKg1	PHKg2	PIM1
PIM2	PIM3	PKA	PKAcb
PKAcg	PKCa	PKCb1	PKCb2
PKCd	PKCepsilon	PKCeta	PKCg
PKCiota	PKCmu/PRKD1	PKCnu/PRKD3	PKCtheta
PKCzeta	PKD2/PRKD2	PKG1a	PKG1b

Kinase Name	Kinase Name	Kinase Name	Kinase Name
PKG2/PRKG2	PKN1/PRK1	PKN2/PRK2	PKN3/PRK3
PLK1	PLK2	PLK3	PLK4/SAK
PRKX	PYK2	RAF1	RET
RIPK2	RIPK3	RIPK4	RIPK5
ROCK1	ROCK2	RON/MST1R	ROS/ROS1
RSK1	RSK2	RSK3	RSK4
SBK1	SGK1	SGK2	SGK3/SGKL
SIK1	SIK2	SIK3	SLK/STK2
SNARK/NUAK2	SNRK	SRMS	SRPK1
SRPK2	SSTK/TSSK6	STK16	STK21/CIT
STK22D/TSSK1	STK25/YSK1	STK32B/YANK2	STK32C/YANK3
STK33	STK38/NDR1	STK38L/NDR2	STK39/STLK3
SYK	TAK1	TAOK1	TAOK2/TAO1
TAOK3/JIK	TBK1	TEC	TESK1
TESK2	TGFBR2	TIE2/TEK	TLK1
TLK2	TNIK	TNK1	TRKA
TRKB	TRKC	TSSK2	TSSK3/STK22C
TTBK1	TTBK2	TXK	TYK1/LTK
TYK2	TYRO3/SKY	ULK1	ULK2
ULK3	VRK1	VRK2	WEE1
WNK1	WNK2	WNK3	YES/YES1
YSK4/MAP3K19	ZAK/MLTK	ZAP70	ZIPK/DAPK3

1. *Supplemental figures and figure legends.*

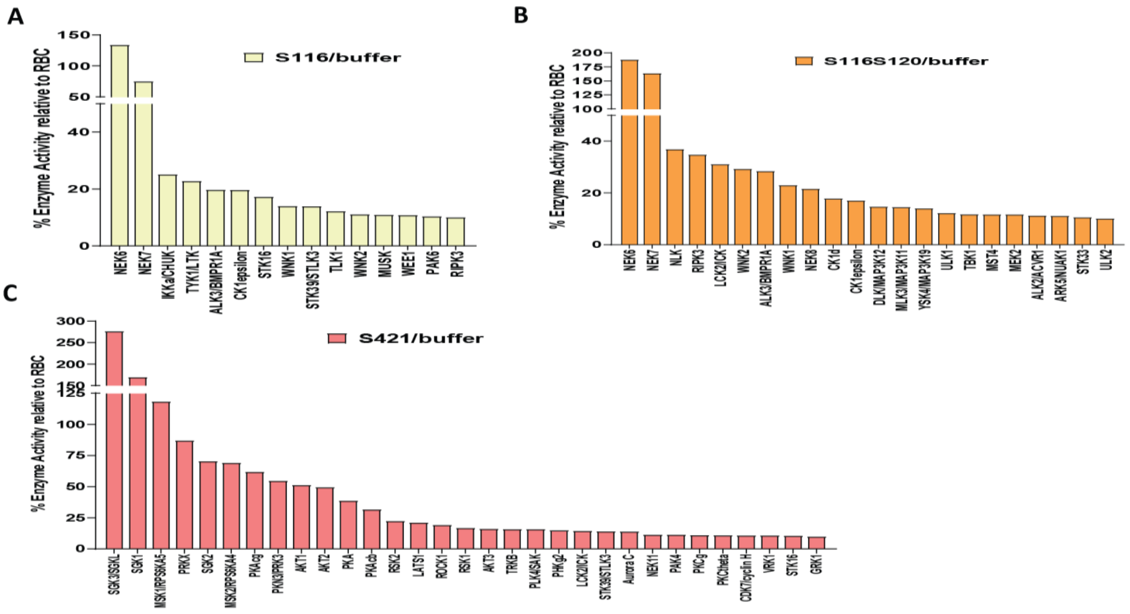

Figure S1. *In vitro kinase assays identified upstream kinases modifying serine 116, 120, and 421 of HTT. In vitro, kinase assay identified kinases targeting HTT peptide-bearing PTMs of HTT other than S1181, S1201, and S2653. Kinases targeted serine 116 (A), 120 (B), and 421 (C).*

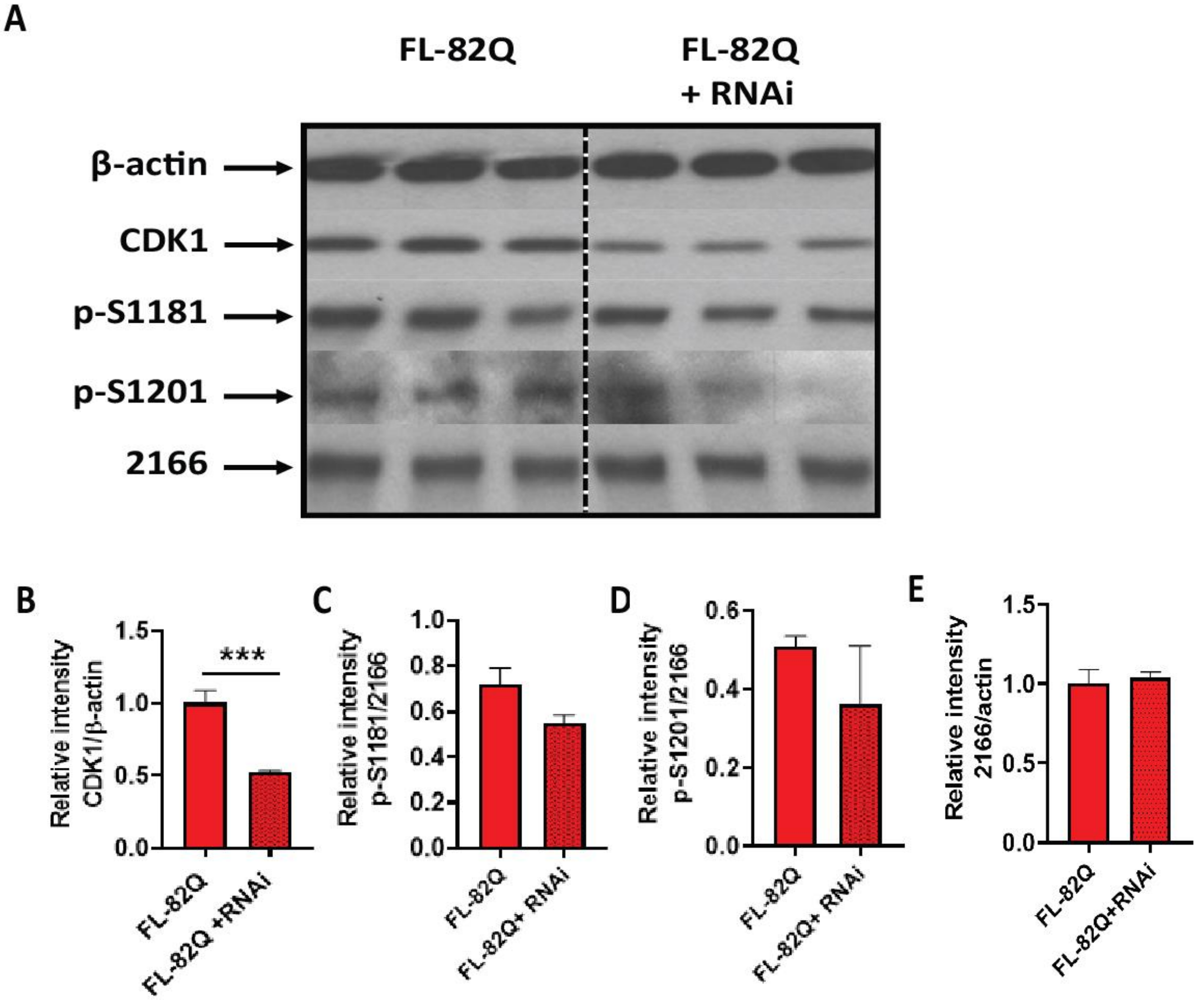

Figure S2. *Knocking down CDK1 did not affect the phosphorylation of S1181 and S1201 of mHTT. HEK293FT cells were co-transfected with a plasmid expressing full-length mutant huntingtin with 82Q (FL-82Q) and siRNA targeting human CDK1 for 24 hours. The expression levels of CDK1, p-S1181-HTT, p-S1201-HTT, and total huntingtin (2166) were detected by western blot. A. Representative images of western blot. B. The quantification of CDK1. C. The quantification of p-S1181. D. The quantification of p-S1201-HTT. E. The quantification of total huntingtin (2166). 2166 represents the anti-HTT antibody (MAB2166). RNAi=siRNA targeting human CDK1. *** p<0.001.*

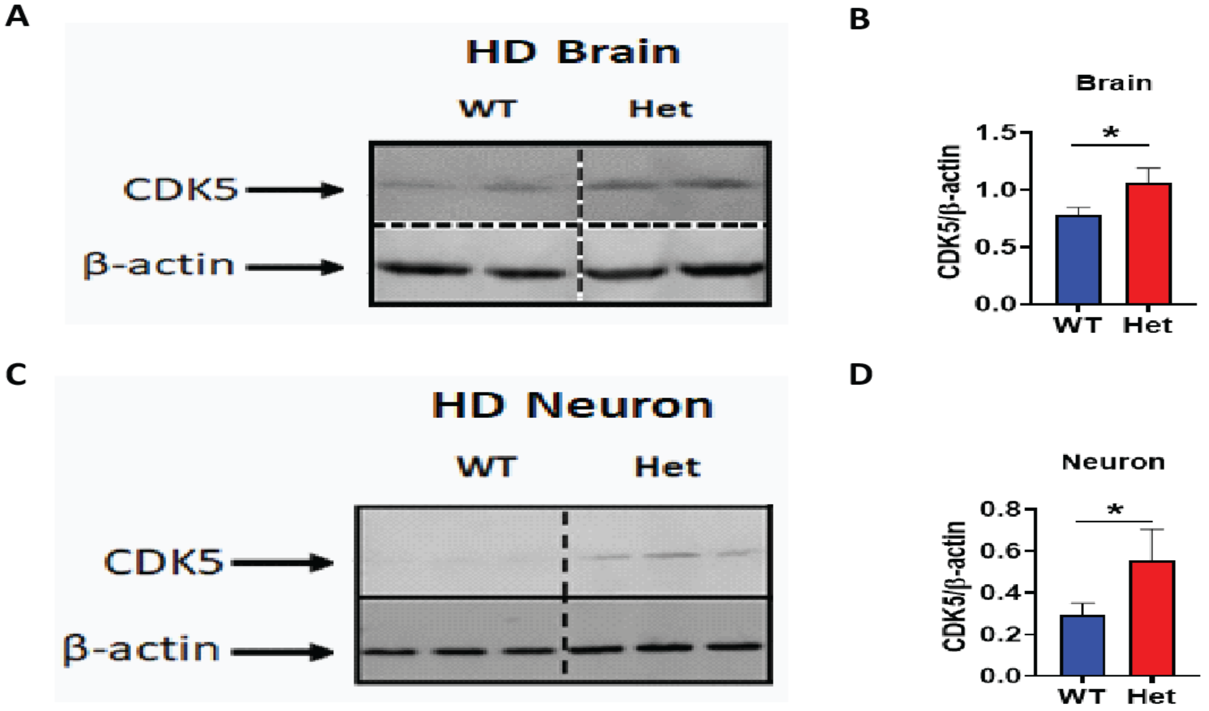

Figure S3. *CDK5 expression was elevated in the brain of HD mouse and primary cultured cortical HD neurons. CDK5 expression was detected in the HD mouse brain and primary cultured neurons isolated from HD mouse using western blot. A. The representative western blot for CDK5 in the striatum of 4-month-old zQ175 HD mouse brain. B. The quantification of CDK5 expression in A. C. The representative western blot for CDK5 in the primary cortical neurons from HD mouse at DIV7. D. The quantification of CDK5 expression in B. * p<0.05.*

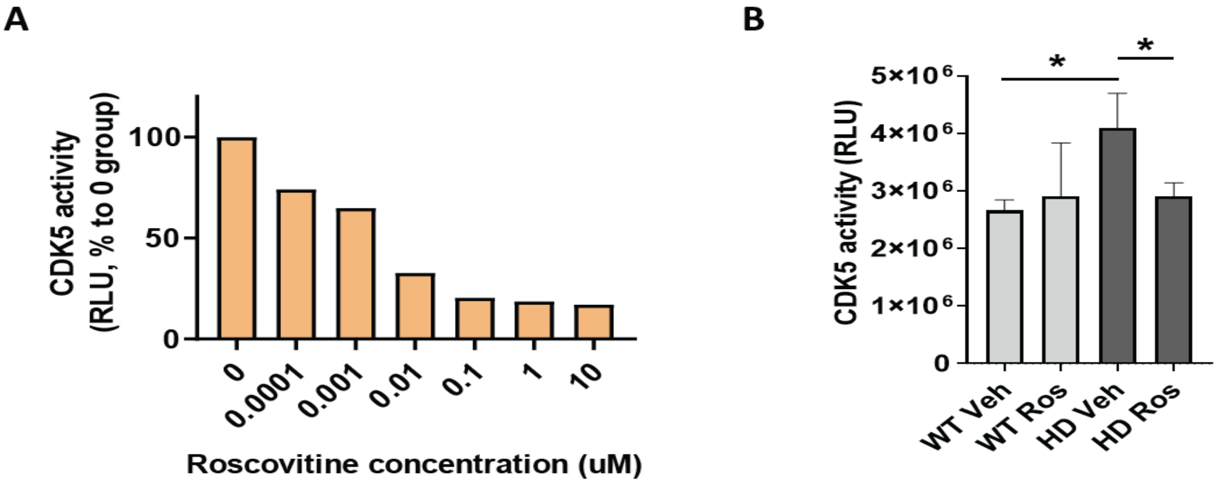

Figure S4. *Roscovitine inhibited the activity of CDK5 in the mouse brain. A. in vitro analysis of roscovitine effect on CDK5 activity, indicating that roscovitine inhibited CDK5 activity in a dose-dependent-manner. B. The CDK5 activity in the brain of mice injected with roscovitine for 3 weeks for both WT and HD mice combined. Veh= Vehicle, Ros=roscovitine. * p<0.05.*
